# Chitooligosaccharide Induces Mitochondrial Biogenesis and Increases Exercise Endurance through the Activation of Sirt1 and AMPK in Rats

**DOI:** 10.1371/journal.pone.0040073

**Published:** 2012-07-11

**Authors:** Hyun Woo Jeong, Si Young Cho, Shinae Kim, Eui Seok Shin, Jae Man Kim, Min Jeong Song, Pil Joon Park, Jong Hee Sohn, Hyon Park, Dae-Bang Seo, Wan Gi Kim, Sang-Jun Lee

**Affiliations:** 1 Amorepacific Corporation Research & Development Center, Bora-dong, Giheung-gu, Yong-in si, Gyeonggi-do, South Korea; 2 College of Physical Education, Kyung Hee University, Seocheon-dong, Giheung-gu, Yong-in city, Gyeonggi-do, South Korea; Instituto Nacional de Cardiologia, Mexico

## Abstract

By catabolizing glucose and lipids, mitochondria produce ATPs to meet energy demands. When the number and activity of mitochondria are not sufficient, the human body becomes easily fatigued due to the lack of ATP, thus the control of the quantity and function of mitochondria is important to optimize energy balance. By increasing mitochondrial capacity? it may be possible to enhance energy metabolism and improve exercise endurance. Here, through the screening of various functional food ingredients, we found that chitooligosaccharide (COS) is an effective inducer of mitochondrial biogenesis. In rodents, COS increased the mitochondrial content in skeletal muscle and enhanced exercise endurance. In cultured myocytes, the expression of major regulators of mitochondrial biogenesis and key components of mitochondrial electron transfer chain was increased upon COS treatment. COS-mediated induction of mitochondrial biogenesis was achieved in part by the activation of silent information regulator two ortholog 1 (Sirt1) and AMP-activated protein kinase (AMPK). Taken together, our data suggest that COS could act as an exercise mimetic by inducing mitochondrial biogenesis and enhancing exercise endurance through the activation of Sirt1 and AMPK.

## Introduction

Chitosan, a linear polysaccharide composed of glucosamine, is produced from chitin, which is the major element of the exoskeleton of crustaceans such as crabs and shrimps. Due to its potential beneficial effects [1]–[5], chitosan has been widely used as a food source and a biomedical agent. Chitooligosaccharide (COS), a mixture of various lengths of glucosamine polymer, is an enzyme-digested product of chitosan. COS exhibits a number of favorable effects including an antimicrobial/antibacterial effect, an anticancer effect, a differentiation-promoting effect, and a wound-healing effect [6]–[13]. Recently COS was suggested to exert a fatigue-reducing effect through the improvement of mitochondrial function and augmentation of mitochondrial biogenesis [14]. However, the underlying mechanism of COS-mediated enhancement of mitochondrial function remains unclear.

Mitochondrial biogenesis is a complex process involving the coordination of a number of proteins derived from both mitochondrial and nuclear genes. Although the underlying mechanism of mitochondrial biogenesis is not clearly understood, peroxisome proliferator-activated receptor gamma coactivator 1 (PGC1) has been implicated as a master regulator of mitochondrial biogenesis through the interaction with nuclear respiratory factor 1 (NRF1) [15], [16]. NRF1 initiates the transcription of genes responsible for mitochondria construction, and PGC1α establishes the network of gene regulation for mitochondrial biogenesis.

Recently, it has been proposed that the expression and activity of PGC1α are regulated by the AMP-activated protein kinase (AMPK) and the silent information regulator two ortholog 1 (Sirt1) [17]–[19]. Both proteins are able to sense the intracellular energy and/or nutritional status. For instance, AMPK is activated under stressful conditions including fasting and osmotic shock to trigger ATP generation by promoting glucose and fatty acid utilization, thereby alleviating metabolic dysfunctions such as hyperglycemia and hyperlipidemia. [20]. Sirt1, a NAD^+^ dependent histone deacetylase, is also activated by fasting and calorie restriction [21], [22]. Similarly to AMPK, Sirt1 is widely associated with energy metabolism [23], [24]. As AMPK and Sirt1 are implicated in mitochondrial function [23], [25], [26], activators of these enzymes could enhance mitochondrial biogenesis and exercise endurance.

In this study, we demonstrated that COS increased the mitochondrial content in skeletal muscle and enhanced exercise endurance in rats. Through the extended exercise endurance, COS-treated rodents exhibited reduced circulating triglyceride and cholesterol level. In differentiated C2C12 myocytes, COS up-regulated the expression and activity of PGC1, one of the essential factors for mitochondrial biogenesis. As a result, COS increased the number of mitochondria and the expression of electron transfer chain (ETC) components. The COS-induced PGC1 activation and mitochondrial biogenesis was mediated by the activation of AMPK and Sirt1. Based on these data, we suggest that COS is an effective metabolic regulator that acts in part by inducing mitochondrial biogenesis and enhancing exercise endurance through the activation of AMPK, Sirt1, and PGC1.

## Results

### COS Increases Mitochondrial Content in Rat Skeletal Muscle to Extend Exercise Endurance

Prior to *in vitro* and *in vivo* experiments, we conducted LC-MS/MS analyses to determine the composition of COS. As shown in **Table 1**, COS consists of the mixture of various length of glucosamine polymers. The average molecular weight of COS is 505.63 Dalton, and the major component of COS is supposed to be a dimer.

**Table 1:** Composition of COS.

#. of glucosamine in polymer	Portion (%)
1	9.32±0.05
2	34.47±1.48
3	23.79±0.01
4	16.13±0.82
5	9.99±1.05
6	4.09±0.06
7	1.81±0.34
8	0.39±0.08
Total	100.00

In a previous study, we demonstrated that COS is able to improve mitochondrial function and improve resistance to fatigue [14]. To be sure that the COS-mediated improvement of mitochondrial function accompanies increased mitochondrial content and enhanced exercise endurance, we administered COS to rats. Remarkably, the wet mass of the soleus muscle (a slow-twitch muscle which contains a large number of mitochondria) was increased upon COS treatment without significant changes in body weight and food intake (**Table 2**). The plasma levels of alanine aminotransferase (ALT) and aspartate aminotransferase (AST) were not changed (**Table 2**), indicating that COS does not induce liver toxicity.

**Table 2:** Biological parameters of COS-treated rats.

Parameters	Vehicle	COS
Initial B.W. (g)	315.3±12.5	305.2±4.4
Final B.W. (g)	330.5±13.7	309.3±3.1^a^
B.W. change (g)	15.2±2.3	4.2±3.7 **^b^**
Daily food intake (g)	33.8±2.5	31.7±2.0
Soleus muscle mass (mg)	241.8±11.4	288.8±36.3^*^
Soleus/B.W. (%)	0.074±0.006	0.093±0.008^***^
Plantaris muscle mass (mg)	706.3±32.4	690.4±32.6
Plantaris/B.W. (%)	0.216±0.013	0.224±0.011
ALT (U/L)	47.3±11.7	43.2±9.3
AST (U/L)	165.8±35.8	171.4±50.7

a P  = 0.19 vs. vehicle; **^b^** P  = 0.08 vs. vehicle; * P<0.05 vs. vehicle; ^***^ P<0.001 vs. vehicle.

**Figure 1:**
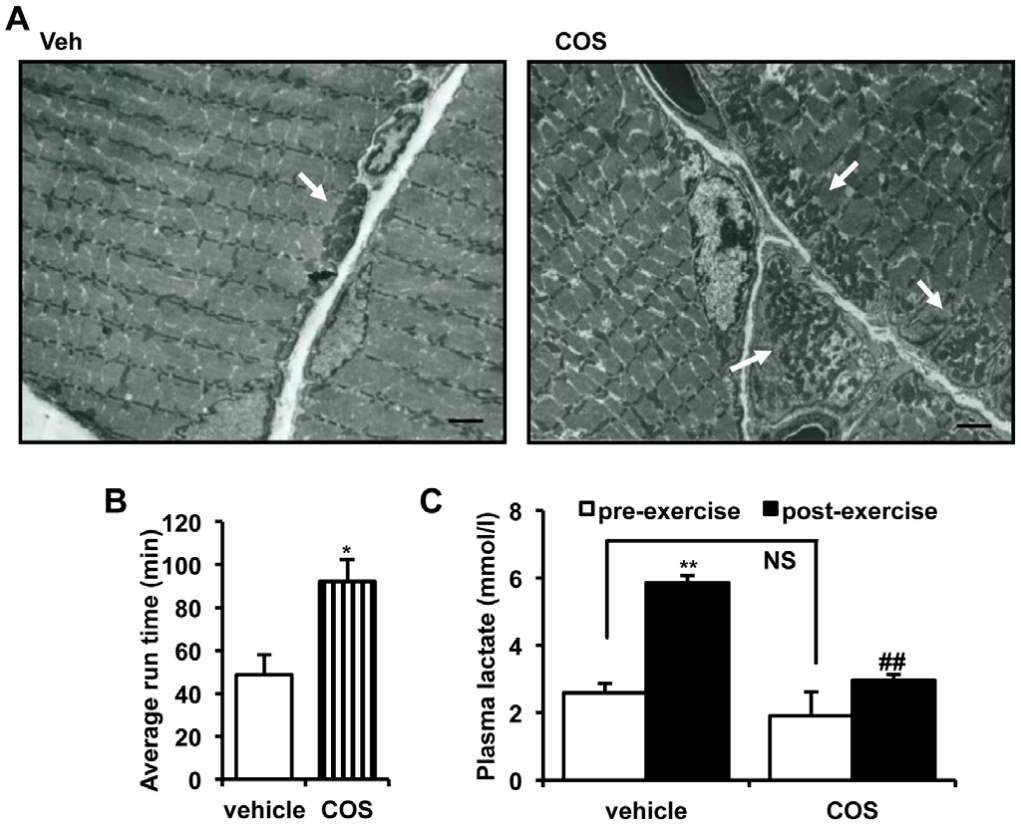
COS increases mitochondrial content *in vivo*. **A**. Electron microscopic image of skeletal muscle. White arrows indicate clusters of mitochondria. Bar  = 2 µM. **B.** Average time of exercise endurance (n  = 6). * P<0.05 vs. vehicle**. C**. Plasma lactate level before and after exercise (n  = 6). ** P<0.01 vs. pre-exercise vehicle (lane 1); ## P<0.01 vs. post-exercise vehicle (lane 2).

Concomitant with the increased soleus muscle weight, mitochondrial content was also increased in COS-administered rats (**Fig. 1A**). Relative mRNA expression level of key genes for mitochondrial biogenesis and energy metabolism, such as PGC1α, NRF1, and carnitine palmitoyl transferase 1b (CPT1b), was augmented in skeletal muscle of COS-administered rats (**Fig. S1**). In agreement with the increased mitochondrial content, COS-administered animals showed extended exercise endurance (**Fig. 1B**), independently of lactate accumulation (**Fig. 1C**), a representative marker of muscle fatigue. Taken together, it is feasible that COS can help increase mitochondrial content and enhance exercise endurance.

### COS Induces Mitochondrial Biogenesis

Through experiments in rodents, we demonstrated that COS augments mitochondrial content in skeletal muscle and enhances exercise endurance. Next, we performed several *in vitro* assays to determine the underlying mechanism of COS-mediated improvement of mitochondrial function.

**Figure 2:**
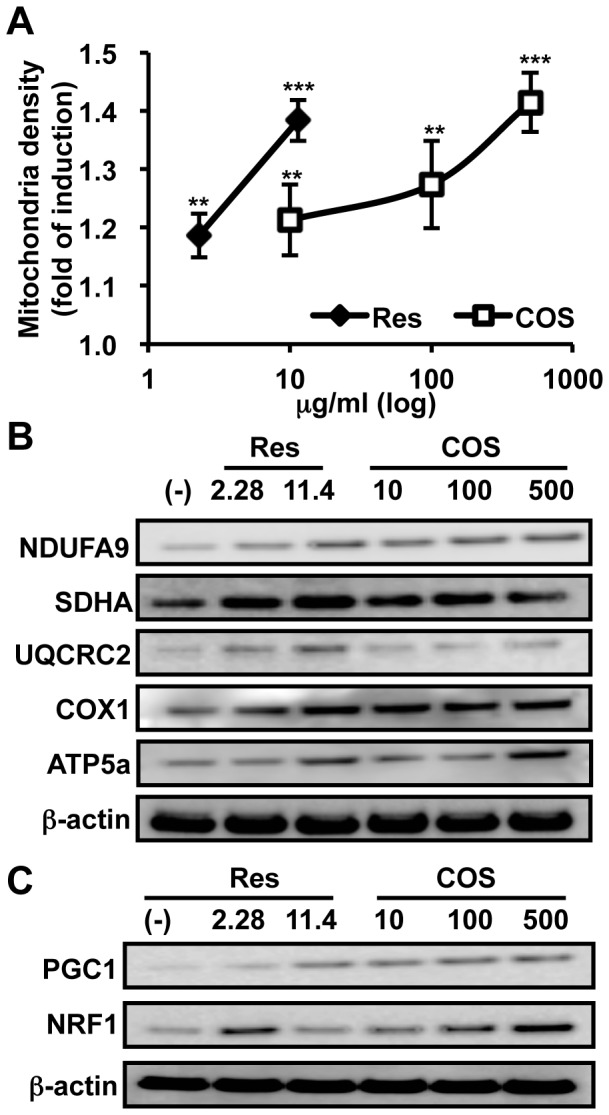
COS increases the mitochondrial content in myocytes. **A**. Differentiated C2C12 cells were treated with resveratrol (Res; 2.28 µg/ml and 11.4 µg/ml) or chitoologosaccharide (COS; 10 µg/ml, 100 µg/ml, and 500 µg/ml) for 24 h. After incubation, the mitochondrial density was measured (n  = 3). **B** and **C**. C2C12 myocytes were treated with Res (10 µM and 50 µM) or COS (10 µg/ml, 100 µg/ml, and 500 µg/ml) for 24 h. After rinsing with PBS, cells were harvested and proteins were blotted with antibodies against NDUFA9, SDHA, UQCRC2, COX1, ATP5a (**B**), or PGC1 and NRF1 (**C**). β-actin was used as a loading control. The densitometry of each band in panel **B** and **C** is shown in **Figure S9A** and S**9B**, respectively.

The energy-generating efficacy of mitochondria is influenced by both mitochondrial function (membrane potential) and quantity (number). To examine whether COS is able to induce mitochondrial biogenesis, we measured mitochondrial density in differentiated C2C12 myocytes following treatment of COS. Similar to the findings *in vivo* (**Fig. 1A**), COS also augmented the mitochondrial density in cultured myocytes (**Fig. 2A**). Concomitant with the increased mitochondrial density, the protein expression of several mitochondrial ETC components including NADH dehydrogenase 1α subcomplex 9 (NDFUA9), succinate dehydrogenase complex subunit A (SDHA), ubiquinol cytochrome c reductase core protein 2 (UQCRC2), cytochrome oxidase subunit 1 (COX1), and ATP synthase mitochondria F1 complex α subunit 1 (ATP5a) was up-regulated in COS-treated C2C12 cells (**Fig. 2B** and **Fig. S9A**).

NRF1 and PGC1 are essential factors for mitochondrial biogenesis by inducing tfam transcription [15]. To elucidate whether COS is able to regulate these transcription regulators, we examined the expression of mitochondrial biogenesis-related genes. As expected, mRNA expression of PGC1α, PGC1β, NRF1, and tfam was augmented upon COS treatment (**Fig. S2**). In agreement with the increase in mRNA expression, the protein level of PGC1 and NRF1 was also increased upon COS treatment (**Fig. 2C** and **Fig. S9B**). Based on these data, we suggest that COS activates a transcriptional cascade responsible for mitochondrial biogenesis.

### COS Activates Sirt1, PGC1, and AMPK

**Figure 3:**
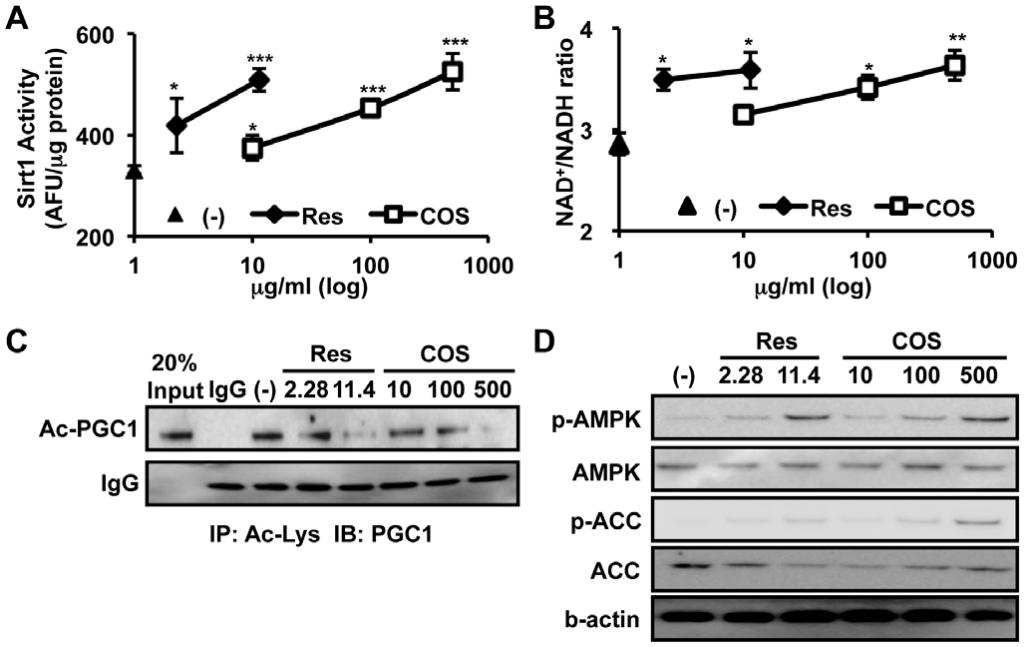
COS activates Sirt1 and AMPK. **A**. Sirt1 activity was examined. Relative Sirt1 activity is shown as a bar graph (n  = 4). **B**. C2C12 myocytes were treated with Res (2.28 µg/ml and 11.4 µg/ml) or COS (10 µg/ml, 100 µg/ml, and 500 µg/ml) for 12 h. The intracellular NAD**^+^**/NADH ratio was calculated (n  = 4). * P<0.05 vs. (-); ** P<0.01 vs. (-); *** P<0.001 vs. (-). **C**. Differentiated C2C12 cells were incubated with Res (10 µM and 50 µM) or COS (10 µg/ml, 100 µg/ml, 500 µg/ml) for 6 h. After incubation, cells were rinsed with PBS and the protein lysates were immunoprecipitated with anti-Ac-Lys antibody and subjected to western blotting. **D**. Differentiated C2C12 myocytes were incubated with Res (11.4 µg/ml) or COS (10 µg/ml, 100 µg/ml, and 500 µg/ml) for 24 h. Then cells were rinsed with PBS two times. Total cell lysates were subjected to western blot analysis using p-AMPK, AMPK, p-ACC, and ACC antibodies. The average band intensities of panel **C** and **D** are shown in **Figure S9C** and S**9D**, respectively.

Sirt1 is reported to activate PGC1 by deacetylation during mitochondrial biogenesis [18], [19], [23], [27]. To determine whether COS could induce mitochondrial biogenesis by activating Sirt1, we measured Sirt1 activity. As shown in **Fig. 3A**, COS increased Sirt1 activity.

As Sirt1 requires NAD^+^ as a substrate, the enzymatic activity of Sirt1 is influenced by the cellular NAD**^+^**/NADH ratio. To determine whether COS regulates the intracellular NAD**^+^**/NADH ratio to activate Sirt1, we measured changes in the cellular NAD**^+^**/NADH ratio. As expected, COS dose-dependently increased the cellular NAD**^+^**/NADH ratio in C2C12 cells (**Fig. 3B**). These data indicate that COS could activate Sirt1 through elevation of cellular NAD^+^/NADH ratio. As a result, the acetylation level of PGC1, which negatively reflects PGC1 activity, was decreased by COS treatment (**Fig. 3C** and **Fig. S9C**).

Similar to Sirt1, AMPK is also proposed to induce mitochondrial biogenesis and enhance fatty acid oxidation [20], [25], [28], [29]. Therefore, we assessed whether COS induced AMPK activation. As expected, COS increased the phosphorylation of Thr_172_ residue of the AMPKα subunit, followed by the phosphorylation of Ser_79_ residue of Acetyl Co-A Carboxylase (ACC) (**Fig. 3D** and **Fig. S9D**), a substrate of AMPK. These data suggest that COS is likely to activate Sirt1 and AMPK, which are responsible for mitochondrial biogenesis.

### COS-mediated Mitochondrial Biogenesis is Sirt1- and AMPK- dependent

**Figure 4:**
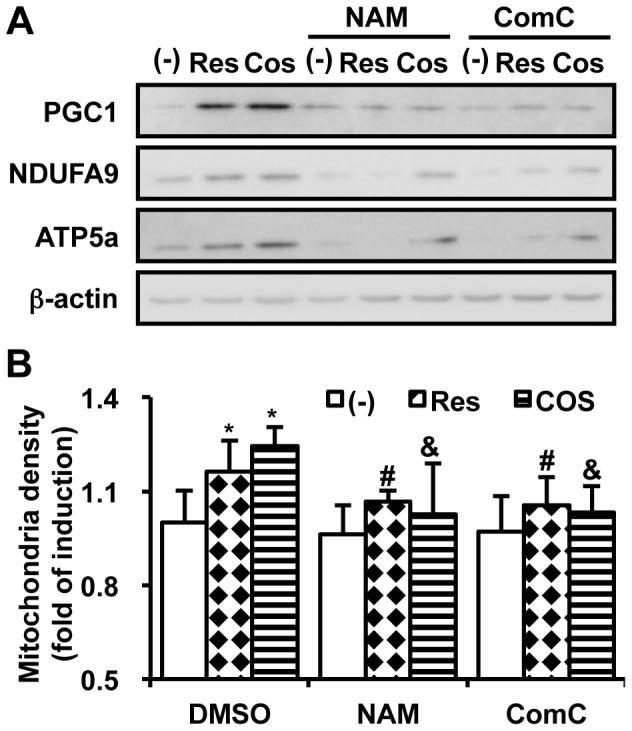
COS requires AMPK and Sirt1 to induce mitochondrial biogenesis. Differentiated C2C12 myocytes were pre-treated with nicotinamide (NAM, 1 mM) or Compound C (ComC; 10 µM) for 2 h prior to incubation with Res (11.4 µg/ml) or COS (500 µg/ml) for 12 h. Cells were rinsed with PBS and harvested for western blotting (**A**) or the measurement of mitochondrial density (n  = 3) (**B**). * P<0.05 vs. DMSO/(-) (lane 1); # P<0.05 vs. Res/(-) (lane 2); & P<0.05 vs. COS/(-) (lane 3). Relative protein expression level of PGC1, NDUFA9, and ATP5a is shown in **Figure S9E**.

We demonstrated that COS activates Sirt1 and AMPK, two regulatory molecules that induce mitochondrial biogenesis. To determine whether Sirt1 and AMPK mediate the effect of COS on mitochondrial biogenesis, we pre-treated nicotinamide (NAM; a Sirt1 inhibitor) and Compound C (ComC; an AMPK inhibitor). As expected, mRNA expression of PGC1α, NRF1, PGC1β, and tfam was abolished by pretreatment with NAM or ComC (**Fig. S3**). Consequently, COS-mediated increment of PGC1 protein level was blocked by NAM and ComC pretreatment (**Fig. 4A** and **Fig. S9E**). The induction of NDUFA9 and ATP5a, mitochondrial ETC components, was also prevented by Sirt1 and AMPK inhibition (**Fig. 4A**). Following Sirt1 or AMPK inhibition, the COS-mediated mitochondrial biogenesis was not observed (**Fig. 4B**).

**Figure 5:**
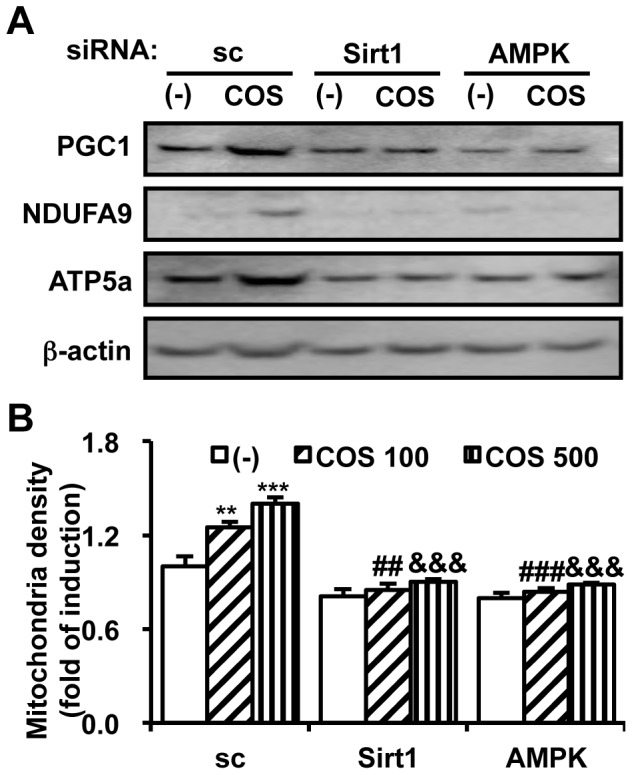
Knockdown of Sirt1 or AMPK expression diminishes the mitochondriogenic effect of COS. C2C12 myotubes were transfected with scramble (sc), Sirt1, and AMPKα siRNA by using Lipofectamine™ 2000 reagent. After transfection, cells were treated with COS (100 µg/ml, and 500 µg/ml) for 12 h. Cells were rinsed with PBS and subjected for western blotting (**A**) or the measurement of mitochondrial density (n  = 3) (**B**). ** P<0.01 vs. sc/(-) (lane 1); *** P<0.001 vs. sc/(-) (lane 1); ## P<0.01 vs. sc/COS 100 (lane 2); ### P<0.001 vs. sc/COS 100 (lane 2); &&& P<0.001 vs. sc/COS 500 (lane 3). Relative expression level of PGC1, NDUFA9, and ATP5a is calculated in **Figure S9G**.

However, it is reported that ComC inhibits HIF-1 activation regardless of AMPK [30], implying that ComC might not act as a specific inhibitor of AMPK. Moreover, NAM actually acts as an activator of Sirt1 in a low dose. Therefore, we also utilized siRNAs to dampen Sirt1 and AMPK activity specifically. Decreased Sirt1 and AMPK expression level by siRNA transfection is shown in **Fig. S4** and **S9F**. While the scramble siRNA (sc) did not affect the effect of COS on mitochondrial gene expression (**Fig. 5A**) and mitochondrial biogenesis (**Fig. 5B**), siRNAs targeting Sirt1 and AMPK blunted the expression of mitochondrial ETC proteins (**Fig. 5A**) and mitochondrial biogenesis (**Fig. 5B**) upon COS treatment, respectively. The expression levels of mitochondria-related proteins in siRNA transfected cells were shown in **Fig. S9G**. Collectively, these data indicate that the activation of Sirt1 and AMPK would be an important step in mediating the effect of COS on mitochondrial biogenesis.

### COS Activates Sirt1 and AMPK in vivo

Sirt1 and AMPK are suggested to be implicated in the signaling pathways to mediate COS-induced mitochondrial biogenesis in cultured myocytes. To confirm whether COS is able to activate these proteins *in vivo*, we examined Sirt1 and AMPK activity in skeletal muscle of COS-administered rats.

**Figure 6:**
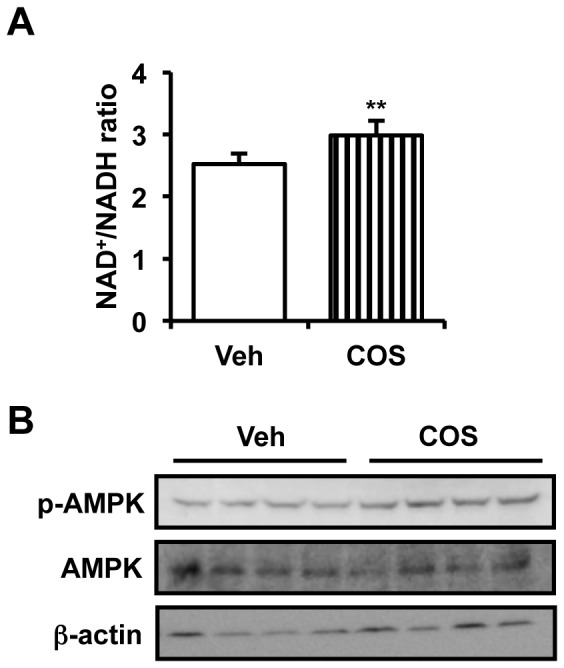
COS activates Sirt1 and AMPK *in vivo*. COS-administered SD rats were sacrificed and proteins from skeletal muscle were subjected to the measurement of cellular NAD^+^/NADH ratio (n  = 6) (**A**) or western blotting (**B**). ** P<0.01 vs. vehicle. Densitometry of p-AMPK was shown in **Figure S9H**.

First, to determine Sirt1 activity, we measured intramuscular NAD^+^/NADH ratio. Similar to *in vitro* experiments (**Fig. 3A**), COS increased NAD^+^/NADH ratio in skeletal muscle (**Fig. 6A**), implying that COS modulates Sirt1 activity *in vivo* as well as *in vitro*. Next, we examined AMPK activity in skeletal muscle by western blotting. As shown in **Fig. 6B** and **Fig. S9H**, COS administration augmented the phosphorylation level of AMPKα catalytic subunit, supposing that COS is able to activate both of Sirt1 and AMPK *in vivo*. Accordingly, our data strongly suggest that COS is a novel activator of Sirt1 and AMPK.

## Discussion

Due to the regulatory role in the energy balance, mitochondria are linked to various diseases including metabolic disorders, Alzheimer’s disease, Parkinson’s disease, cancer and aging [31]–[37]. Therefore, the maintenance of mitochondrial function plays an essential role in sustaining a healthy life. Previously we demonstrated that COS is able to improve mitochondrial function and resistance to fatigue in mice [14]. In this study, we analyzed the composition of COS and identified the molecular mechanism of COS-mediated mitochondrial biogenesis. COS initiates a general process of mitochondrial biogenesis. COS activated AMPK, Sirt1, and PGC1 to induce mitochondrial biogenesis in myocytes. In rodents, COS increased both of mitochondrial content in skeletal muscle and enhanced exercise endurance. Interestingly, chitosan (average molecular weight of 9,000 Dalton) failed to mimic the mitochondriogenic effect of COS (data not shown), indicating that the induction of mitochondrial biogenesis is a unique property of COS.

PGC1, which is activated during exercise, is an important inducer of mitochondrial biogenesis and muscle fiber type change [16], [38]–[40], which are directly associated with energy metabolism. It has been reported that decreased expression of PGC1α and reduced mitochondrial DNA content were observed in type 2 diabetes patients [41]. PGC1β is also implicated in mitochondrial fusion, which is directly associated with mitochondrial function [38]. These results emphasize the important role of PGC1 in the maintenance of mitochondrial function and energy metabolism. As a master regulator of mitochondrial biogenesis, NRF1/PGC1 complex regulates mitochondrial biogenesis-related genes (e.g. tfam), and tfam is responsible for the induction of mitochondrial genes [42]. Therefore, it would be reasonable to assume that PGC1 is also able to regulate mitochondrial gene expression. In the present study, we found a novel substance that is able to regulate both of expression and activity of PGC1. These data imply that COS could be a novel inducer of mitochondrial biogenesis and an enhancer of energy metabolism through the activation of the PGC1 pathway.

The activity of PGC1 is primarily regulated by Sirt1 and AMPK [17]–[19], [28], [43], which also regulate energy metabolism and mitochondrial biogenesis. In our experiments, blockade of Sirt1 and AMPK activity with chemical inhibitors and specific siRNAs, which are more specific than chemical inhibitors (NAM and ComC), abrogated the effect of COS on mitochondrial biogenesis. In the presence of these molecules, COS failed to increase the expression of mitochondrial ETC components and facilitate mitochondrial biogenesis. Although the underlying mechanism for the COS-induced activation of Sirt1 and AMPK requires further studies, our data illustrate that the activation of Sirt1 and AMPK is important for the induction of mitochondrial biogenesis upon COS treatment.

Following enhanced density and activity of muscle mitochondria in rodents, we demonstrated that COS extended exercise endurance without excess lactate accumulation. Moreover, enzymatic activities of lactate dehydrogenase (LDH) and creatine kinase (CK), markers of muscle damage, were not increased in COS group after endurance exercise, compared with those of vehicle group (**Fig. S5**), indicating that COS also raised cellular stress resistance. Our results suggest that COS delayed muscle fatigue and enhanced exercise endurance, analogously to other mitochondrial enhancers [44]–[48].

The plasma level of lipids is closely associated with metabolic syndromes such as hyperlipidemia and insulin resistance [49], [50]. It has been reported that regular exercise, which promotes lipid utilization, reduces plasma lipid parameters and improves insulin sensitivity [51]–[55]. In our experiments, COS-administered animals exhibited reduced plasma triglyceride (TG) and cholesterol levels (**Fig. S6**). Furthermore, COS augmented fatty acid oxidation in cultured myotubes (**Fig. S7**). Therefore, it is likely that COS-administered rats might prefer lipids than glucoses to extend exercise endurance. According to these data, it would be reasonable that COS might be helpful to alleviate hyperlipidemia and its detrimental effects through lipid utilization and exercise extension in skeletal muscle.

It would be interesting to identify the active compound of COS. By using LC-MS/MS analysis, we succeeded in analyzing the composition of COS and in obtaining several low-molecular weight (LMW) form of COS (from dimer to tetramer). However, other components (pentamer or higher molecular weight) could not be separated because they were eluted almost at the same time (data not shown). Due to these technical problems, we had to test only the LMW form of COS. Surprisingly, LMW form of COS did not induce Sirt1 activation (**Fig. S8A**). Moreover, they also failed to phosphorylate AMPK and to increase the expression of PGC1 and NDUFA9 (**Fig. S8B** and **9I**), genes which are responsible for mitochondria. In spite of the technical limitation, we found that the LMW form of COS does not possess mitochondriogenic effect in cultured myotubes. We assume that the active component of COS might be in the high-molecular weight (HMW) fraction. And that would be the reason why the effect of COS on mitochondrial biogenesis was lesser than that of Res in a higher dose. Res is a pure chemical, whereas COS is a mixture of active (HMW) and inactive (LMW) glucosamine. The proportion of HMW, which is considered to contain an active compound, is less than 20% of total amount of COS (**Table 1**), thereby limiting the activity of COS mixture. Further studies with advanced purification methods will allow us to identify the active component of COS in a HMW fraction and enhance the activity of COS.

In conclusion, COS activated AMPK and increased the cellular NAD**^+^**/NADH ratio to induce Sirt1 activation. The activation of AMPK and Sirt1 increased the expression and activity of PGC1 and augmented the expression of mitochondrial genes. As a result of activation of AMPK, Sirt1, and PGC1, COS facilitated mitochondrial biogenesis. In rodents, the administration of COS significantly increased intramuscular mitochondrial content, resulting in enhanced exercise endurance and reduced plasma lipid profiles. Collectively, our data suggest that COS could enhance exercise endurance by promoting mitochondrial biogenesis.

## Materials and Methods

### Preparation and Analysis of COS

COS was produced by Bioland Korea Co. Briefly, COS was produced from chitosan by enzyme digestion, followed by deacetylation of chitin, as described previously [56]. After enzyme removal, mixture of chitosan fragments was dissolved with 0.1% lactic acid. To identify the composition of COS, COS was analyzed by LC-MS/MS (Agilent 6410 LC/MASS and Agilent 1200 HPLC, Agilent Technologies) analysis. The reaction conditions are provided in **Table S1**.

### Animal Experiment and Exercise Endurance Test

All animal experiments were approved by the Amorepacific Institutional Animal Care and Use Committee (AP10-188-FR003) and adhere to the OECD guidelines. Thirty-nine-week-old female Sprague-Dawley rats were purchased from Central Lab. Animal Inc. and fed normal chow *ad libitum*. For COS administration, COS (0.5% w/w) was mixed with normal chow and provided to rats for 6 w (n  = 12), and the control group (vehicle) was provided normal chow only (n  = 12). Rats were placed on the treadmill (0° slope and 15 m/min for 15 min) for treadmill adaptation every other week.

Half of each group was sacrificed prior to the exercise endurance test to measure the pre-exercise plasma profiles including ALT, AST, TG, total cholesterol, lactate, and FFA. To examine exercise endurance, rats were subjected to a run on the treadmill (7**°** slope and 20 m/min). At the end of the treadmill, a weak electric stimulator was located to encourage rats to run forward, as recommended previously [57]. Exercise time was measured until the rat failed to move on the treadmill despite electrical stimulation. After exercise, the rats were sacrificed and the skeletal muscles were prepared. Skeletal muscles were immediately freeze-sectioned for electron microscope imaging. Plasma samples were taken for further analyses.

### Reagents

Compound C and nicotinamide were purchased from Calbiochem and Sigma, respectively. The antibodies for phospho-AMPK, AMPK, phospho-ACC, ACC, and β-actin were obtained from Cell Signaling. The antibodies for PGC1, NRF1, NDUFA9, SDHA, UQCRC2, COX1, ATP5a, and acetylated lysine and siRNAs targeting Sirt1 and AMPK and non-specific siRNA (scramble) were purchased from Santa Cruz Biotechnology Inc. Plasma ALT and AST measuring kits were purchased from Bayer.

### Cell Culture

C2C12 cells were purchased from American Type Culture Collection. Cells were grown in DMEM (Lonza) medium supplemented with 10% FBS (Hyclone). All media were supplemented with 100 units/ml of penicillin and 100 mg/ml of streptomycin (Lonza), and all cells were grown at 37^○^C in a humidified atmosphere containing 5% CO_2_. For myoblast differentiation, confluent C2C12 cells were maintained with DMEM supplemented with 2% horse serum (Hyclone) for 7 d. During differentiation, the medium was changed daily.

### Measurement of Mitochondrial Density

The content of mitochondria was measured by using MitoTracker® Green FM (Invitrogen) reagent following the manufacturer’s protocol. Briefly, cells were rinsed with PBS twice and 50 nM of diluted MitoTracker® Green FM was added to each well for 30 min. Fluorescence of MitoTracker® Green FM was measured to determine the mitochondrial content by using TECAN M200pro fluorometric plate reader (Tecan).

### Western Blotting

An equal amount of protein (60 µg) from each sample was separated on NuPAGE® Novex® Tris-Acetate gels (Invitrogen) and transferred to membranes using an iBlot® dry transfer device (Invtirogen). Blots were blocked with 5% non-fat milk (Sigma) in tris-buffered saline (Bio-Rad) supplemented with 0.1% Tween-20 (Sigma) (TBST) at room temperature (RT) for 15 min. After blocking, the blots were incubated with primary antibodies dissolved in TBST/5% BSA (Sigma) with gentle shaking at RT for 2 h. After washing with TBST three times, the membranes were hybridized with horseradish peroxidase-conjugated secondary antibodies (Bio-Rad) in 5% non-fat milk dissolved in TBST at RT for 2 h and washed three times with TBST. The blots were then incubated with enhanced chemiluminescence reagents (Animal Genetics Inc.) and analyzed with a LAS-3000 imaging system (Fujifilm).

### Sirt1 Activity Assay and Measurement of Cellular NAD^+^/NADH Ratio

A Sirt1 activity assay was performed using the SIRT1 Fluorimetric Drug Discovery Kit (Biomol International), as previously described [58]. Briefly, total protein (25 µg) and acetylated substrate (Fluor-de-Lys-Sirt1 substrate, 100 µM) were incubated at 37^○^C in the presence of NAD (100 µM) for 30 min. After incubation, de-acetylated substrates were detected on a TECAN M200pro fluorometric plate reader.

The intracellular NAD^+^/NADH ratio was assessed using an NAD/NADH assay kit (Biovision) with the provided protocol. Briefly, cells or tissues were lysed with 400 µl of NADH/NAD extraction buffer by freezing/thawing. Extracts were vortexed for 10 s and centrifuged at 14,000 rpm for 5 min. Supernatants containing NAD^+^ and NADH were transferred into a new microcentrifuge tube. To determine the level of NADH, samples were incubated at 60^○^C for 30 min to disrupt NAD^+^. NADt (total NAD**^+^** and NADH) and NADH samples were mixed with an NAD cycling enzyme that converts NAD**^+^** to NADH at RT for 5 min and incubated with the NADH developing agent at RT for 1 h. NADH levels were determined by measuring the absorbance at 450 nm. NAD^+^ levels were calculated by subtracting NADH from NADt.

### Immunoprecipitation

Equal amounts of the protein lysates (500 µg) were incubated with 1 µg of anti-acetylated lysine antibody at RT for 2 h. For the negative control, protein lysate was incubated with normal mouse IgG (Santa Cruz). After incubation, each lysate was added to protein A/G plus agarose (Santa Cruz) and further incubated with gentle shaking at RT for 2 h. Lysates were centrifuged and the supernatants were discarded. Pellets containing precipitated proteins were sampled, separated, and analyzed by western blotting.

### siRNA Transfection

For knockdown of AMPKα and Sirt1, differentiated C2C12 myocytes were transfected with mouse AMPKα siRNA (targeting both of AMPKα1 and AMPKα2) or mouse Sirt1 siRNA using Lipofectamine™ 2000 reagent (Invitrogen) and incubated in Opti-MEM (Invitrogen) medium for 4 h, as the manufacturer’s protocol. After transfection, the medium was replaced with fresh DMEM medium containing 10% FBS.

### Statistical Analyses

Results are representative of data from at least three repetitive experiments. All data points represent the average of triplicate samples. Error bars represent the SD (*in vitro*) and SEM (*in vivo*). The values calculated from one-way ANOVA (followed by two-tailed unpaired Student’s *t*-test) P<0.05 were interpreted as statistically significant.

## Supporting Information

Figure S1 **COS increases mRNA expression of mitochondria-related genes.** mRNAs were isolated from skeletal muscle of animals described in **Figures 1** and **5** and RNAs were isolated and cDNAs were synthesized using Trizol™ Reagent (Invitrogen) and RevertAid™ First Strand cDNA Synthesis Kit (Fermentas), respectively. The relative mRNA level of PGC1a, NRF1, and CPT1b was measured by qPCR and normalized to GAPDH (n  = 6). * P<0.05 vs. vehicle; ** P<0.01 vs. vehicle. The primer sequences are listed in **Table S2**.
(TIF)

Figure S2 **COS increases mRNA expression of mitochondrial transcription factors.** Differentiated C2C12 cells were treated with Res (2.28 µg/ml and 11.4 µg/ml) or COS (10 µg/ml, 100 µg/ml, and 500 µg/ml) for 24 h and washed with PBS two times, and RNAs were isolated and cDNAs were synthesized. Relative mRNA expression of PGC1α (**A**), PGC1β (**B**), NRF1 (**C**), and tfam (**D**) was measured by using qPCR and normalized to GAPDH (n  = 3). * P<0.05 vs. (-); ** P<0.01 vs. (-).
(TIF)

Figure S3 **AMPK and Sirt1 inhibitor prevents COS-induced mRNA expression of mitochondrial biogenesis-related genes.** Differentiated C2C12 myocytes were pre-treated with nicotinamide (NAM, 1 mM) or Compound C (ComC; 10 µM) for 2 h prior to incubation with Res (11.4 µg/ml) or COS (500 µg/ml) for 12 h. Cells were rinsed with PBS and harvested for qPCR analysis. cDNAs were subjected to qPCR analysis to detect the relative mRNA expression of PGC1α (**A**), PGC1β (**B**), NRF1 (**C**), and tfam (**D**) (n  = 3). * P<0.05 vs. DMSO-treated (-) (lane 1); ** P<0.01 vs. DMSO-treated (-); *** P<0.001 vs. DMSO-treated (-); # P<0.05 vs. DMSO-treated Res; ## P<0.01 vs. DMSO-treated Res; & P<0.05 vs. DMSO-treated COS; && P<0.01 vs. DMSO-treated COS.
(TIF)

Figure S4 **Knockdown effect of Sirt1 and AMPKα siRNAs in myocytes.** Differentiated C2C12 cells were transfected with non-specific siRNA (sc; 100 pmol), Sirt1 siRNA (Sirt1; 100 pmol), or AMPKα siRNA (AMPK; 100 pmol). After transfection, cells were washed with PBS twice and harvested. **A**. mRNAs were isolated and cDNA was synthesized. The relative mRNA level of Sirt1 and AMPK was measured by qPCR and normalized to GAPDH (n  = 2). * P<0.05 vs. control siRNA. **B**. proteins were subjected to Western Blot to detect the expression level of Sirt1 or AMPKα. β-actin expression was measured as a loading control. Average band intensity is shown in **Figure S9F**. Based on the changes of mRNA and protein expression level after siRNA transfection, the estimated knockdown efficiency of each siRNA is approximately 50% (Sirt1) and 60% (AMPK), respectively.
(TIF)

Figure S5 **COS does not cause excess fatigue in spite of enhanced exercise endurance.** Rats after endurance exercise were sacrificed and plasma LDH (**A**) and CK (**B**) activity was measured by using LDH and CK measuring kit (Bayer), respectively (n  = 6). No statistical significance was observed among groups.
(TIF)

Figure S6 **COS reduces plasma TG and cholesterol levels.** Graphs show plasma profiles of TG (**A**) and cholesterol (**B**) of before (white bar) and after (black bar) exercise (n  = 6). * P<0.05 vs. pre-exercise vehicle (lane 1); # P<0.05 vs. post-exercise vehicle (lane 2).
(TIF)

Figure S7 **COS augments fatty acid oxidation.** Differentiated C2C12 myocytes were treated with Res (11.4 µg/ml) or COS (10 µg/ml, 100 µg/ml, and 500 µg/ml) for 24 h (n  = 10 in each group). After incubation, cells were rinsed with PBS and incubated in α-MEM (Lonza) containing 0.1 mmol/l palmitate (9,10-[^3^H]palmitate, 5 mCi/ml, PerkinElmer Life, Boston, MA) and 2% BSA for 24 h. The medium was then precipitated with an equal volume of 10% trichloroacetic acid (Sigma) by centrifugation at 12,000 rpm for 10 min. The supernatants were transferred to open 1.5 ml microcentrifuge tubes, placed in a scintillation vial containing 0.5 ml water, and incubated at 55°C for 12 h. After the removal of tubes containing the precipitated medium, the ^3^H_2_O contents were measured in a scintillation counter (PerkinElmer Life) in the presence of an enhancer solution (PerkinElmer Life). * P<0.05 vs. (-).
(TIF)

Figure S8 **Small components of COS do not activate Sirt1 and AMPK.** **A**. Sirt1 activity using small constituents of COS (LOW; monomer to tetramer) (n  = 3). * P<0.05 vs. (-); ** P<0.01 vs. (-); *** P<0.001 vs. (-). **B**. Differentiated C2C12 myocytes were treated with COS (500 µg/ml), glucosamine-lactate (1; 500 µg/ml), or mixture of dimer, trimer, and tetramer (2–4; 500 µg/ml) for 24 hours. Proteins were separated and hybridized with p-AMPK, PGC1, NDUFA9, and β-actin antibodies respectively. The band intensity of each band is shown in **Figure S9I**.
(TIF)

Figure S9 **Densitometry of Western Blot results.** Band intensity of each band in the **Figure 2B** (**A**), **Figure 2C** (**B**), **Figure 3C** (**C**), **Figure 3D** (**D**), **Figure 4A** (**E**), **Figure S4B** (**F**), **Figure 5A** (**G**), **Figure 6B** (**H**), and **Figure S8B** (**I**) was measured by Multiguage software (Fujifilm) and showed in bar graphs. * P<0.05 vs. (-); ** P<0.01 vs. (-); *** P<0.001 vs. (-).
(TIF)

Table S1 **Reaction condition of LC-MS/MS analysis.** The composition of COS was analyzed by conducting LC-MS/MS analysis. The brief condition of analysis is shown.
(DOC)

Table S2 **Sequences of qPCR primers.** The qPCR primers for mitochondria-related genes (PGC1α, PGC1β, NRF1, tfam, and CPT1b) were used to examine the effect of COS on mitochondrial biogenesis. The qPCR primers for AMPKα1 and Sirt1 were used to determine the knock-down efficiency of AMPKα and Sirt1 siRNAs, respectively. GAPDH primer was used to normalize the relative mRNA expression of each gene. All qPCR primers were purchased from Bioneer Co.
(DOC)
