# Supplementary figures and images for: Chitooligosaccharide Induces Mitochondrial Biogenesis and Increases Exercise Endurance through the Activation of Sirt1 and AMPK in Rats
Source: PLoS One. 2012 Jul 11;7(7):e40073. doi: 10.1371/journal.pone.0040073 (PMC3394803; doi:10.1371/journal.pone.0040073)

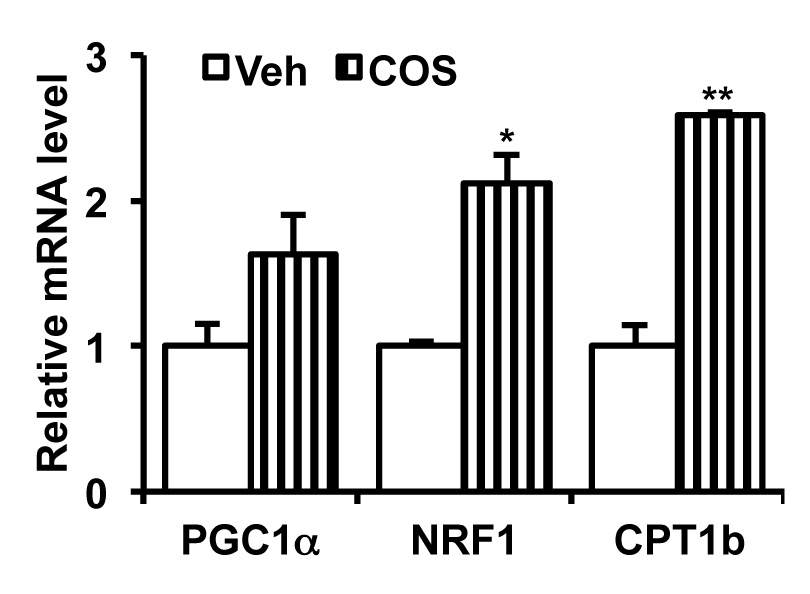

Supplement: Figure S1 — COS increases mRNA expression of mitochondria-related genes. mRNAs were isolated from skeletal muscle of animals described in Figures 1 and 5 and RNAs were isolated and cDNAs were synthesized using Trizol™ Reagent (Invitrogen) and RevertAid™ First Strand cDNA Synthesis Kit (Fermentas), respectively. The relative mRNA level of PGC1a, NRF1, and CPT1b was measured by qPCR and normalized to GAPDH (n = 6). * P<0.05 vs. vehicle; ** P<0.01 vs. vehicle. The primer sequences are listed in Table S2. (TIF) [file pone.0040073.s001.tif]

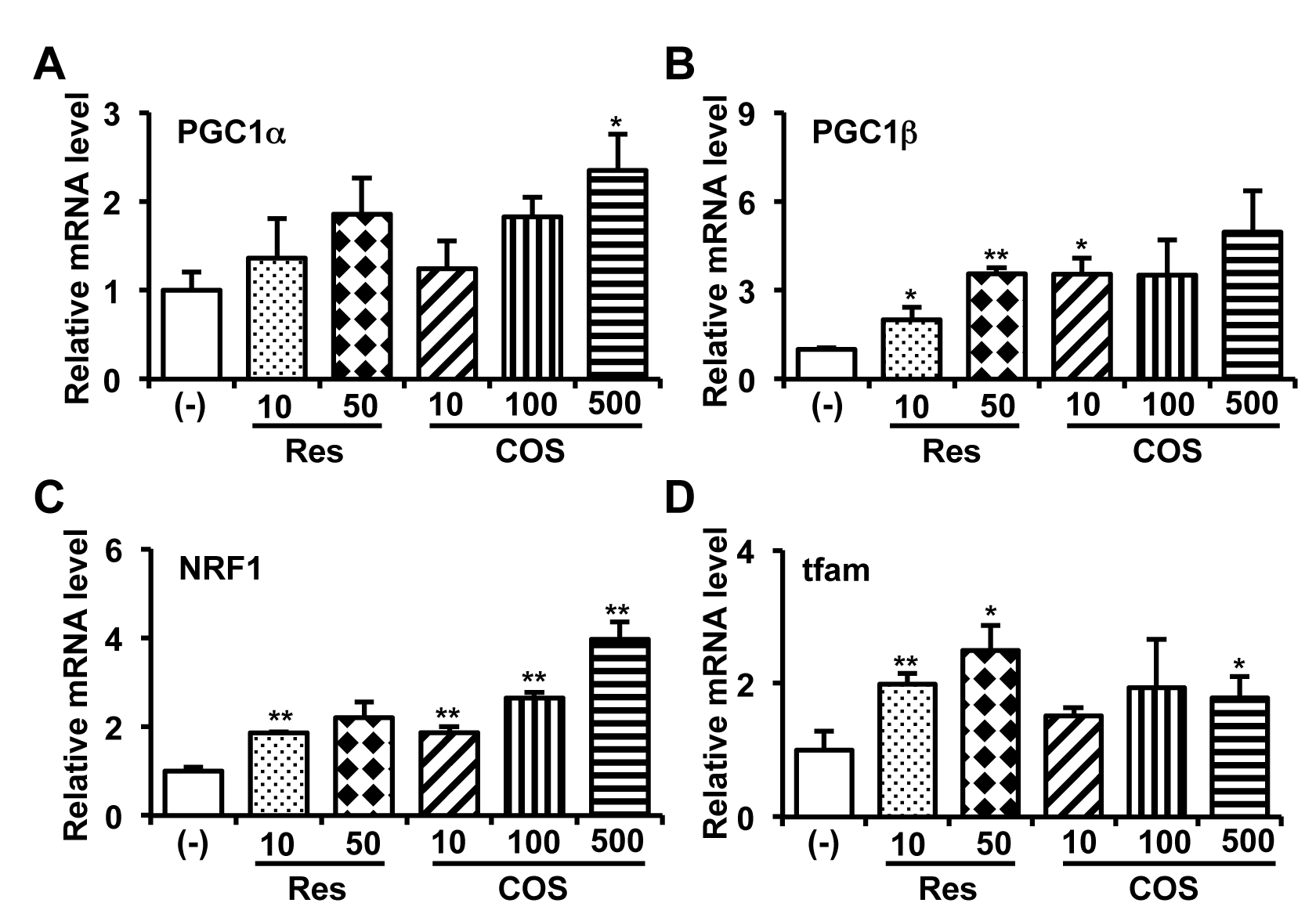

Supplement: Figure S2 — COS increases mRNA expression of mitochondrial transcription factors. Differentiated C2C12 cells were treated with Res (2.28 µg/ml and 11.4 µg/ml) or COS (10 µg/ml, 100 µg/ml, and 500 µg/ml) for 24 h and washed with PBS two times, and RNAs were isolated and cDNAs were synthesized. Relative mRNA expression of PGC1α (A), PGC1β (B), NRF1 (C), and tfam (D) was measured by using qPCR and normalized to GAPDH (n = 3). * P<0.05 vs. (-); ** P<0.01 vs. (-). (TIF) [file pone.0040073.s002.tif]

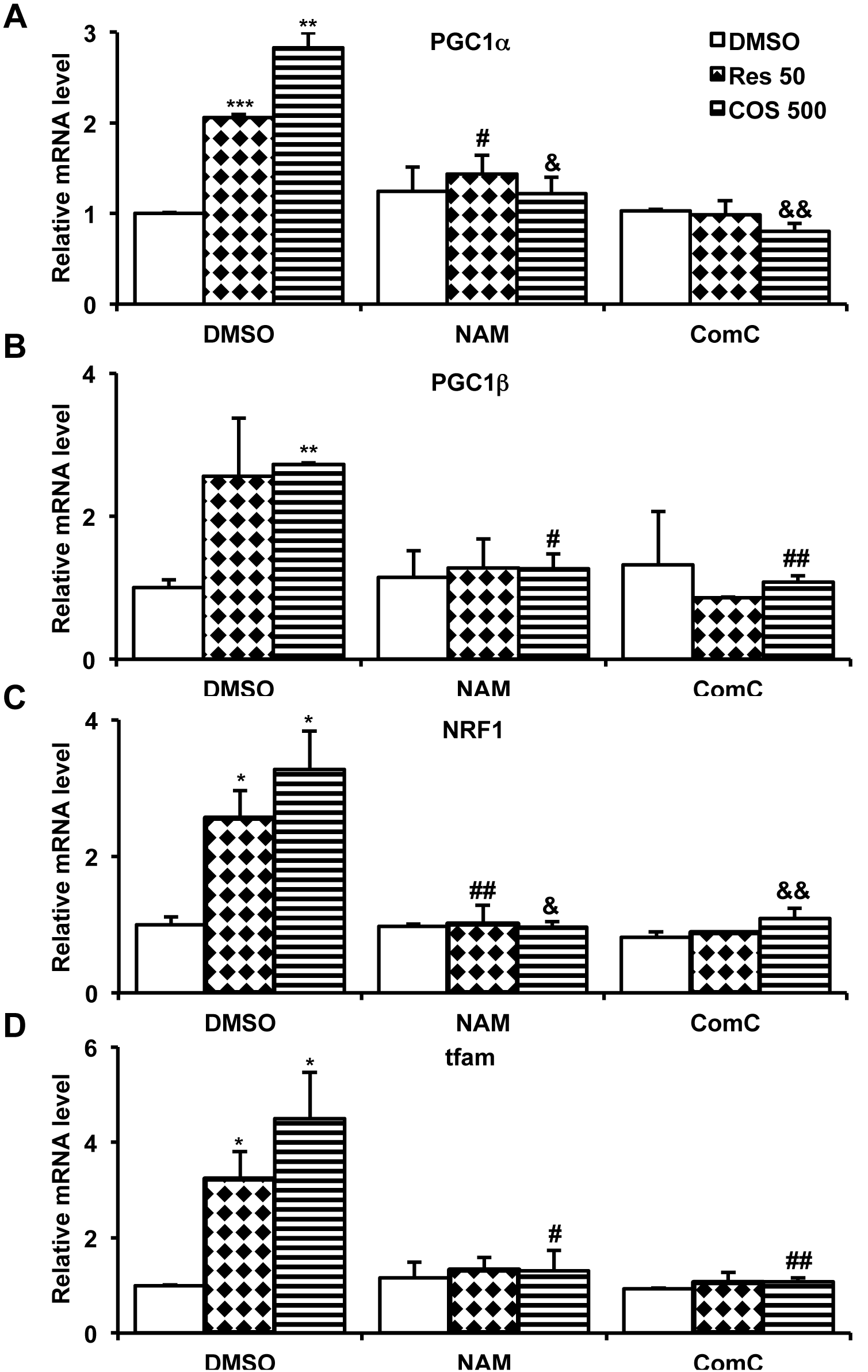

Supplement: Figure S3 — AMPK and Sirt1 inhibitor prevents COS-induced mRNA expression of mitochondrial biogenesis-related genes. Differentiated C2C12 myocytes were pre-treated with nicotinamide (NAM, 1 mM) or Compound C (ComC; 10 µM) for 2 h prior to incubation with Res (11.4 µg/ml) or COS (500 µg/ml) for 12 h. Cells were rinsed with PBS and harvested for qPCR analysis. cDNAs were subjected to qPCR analysis to detect the relative mRNA expression of PGC1α (A), PGC1β (B), NRF1 (C), and tfam (D) (n = 3). * P<0.05 vs. DMSO-treated (-) (lane 1); ** P<0.01 vs. DMSO-treated (-); *** P<0.001 vs. DMSO-treated (-); # P<0.05 vs. DMSO-treated Res; ## P<0.01 vs. DMSO-treated Res; & P<0.05 vs. DMSO-treated COS; && P<0.01 vs. DMSO-treated COS. (TIF) [file pone.0040073.s003.tif]

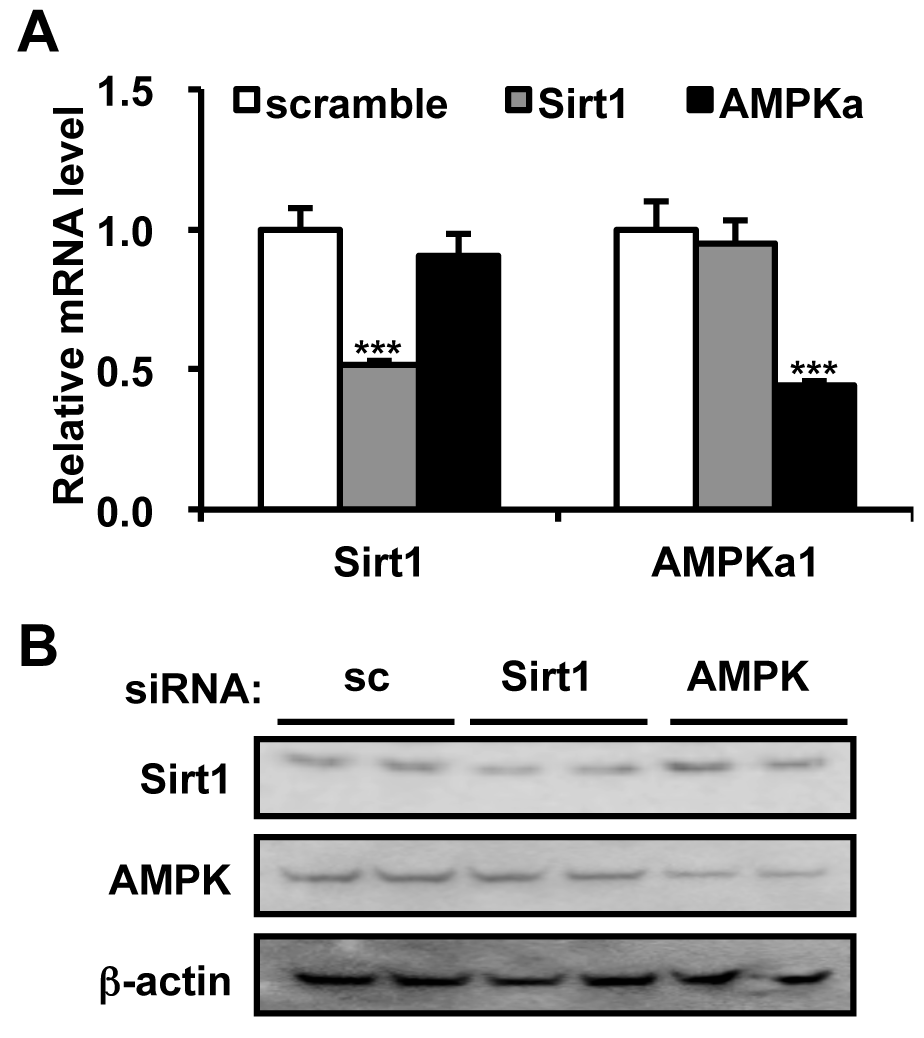

Supplement: Figure S4 — Knockdown effect of Sirt1 and AMPKα siRNAs in myocytes. Differentiated C2C12 cells were transfected with non-specific siRNA (sc; 100 pmol), Sirt1 siRNA (Sirt1; 100 pmol), or AMPKα siRNA (AMPK; 100 pmol). After transfection, cells were washed with PBS twice and harvested. A. mRNAs were isolated and cDNA was synthesized. The relative mRNA level of Sirt1 and AMPK was measured by qPCR and normalized to GAPDH (n = 2). * P<0.05 vs. control siRNA. B. proteins were subjected to Western Blot to detect the expression level of Sirt1 or AMPKα. β-actin expression was measured as a loading control. Average band intensity is shown in Figure S9F. Based on the changes of mRNA and protein expression level after siRNA transfection, the estimated knockdown efficiency of each siRNA is approximately 50% (Sirt1) and 60% (AMPK), respectively. (TIF) [file pone.0040073.s004.tif]

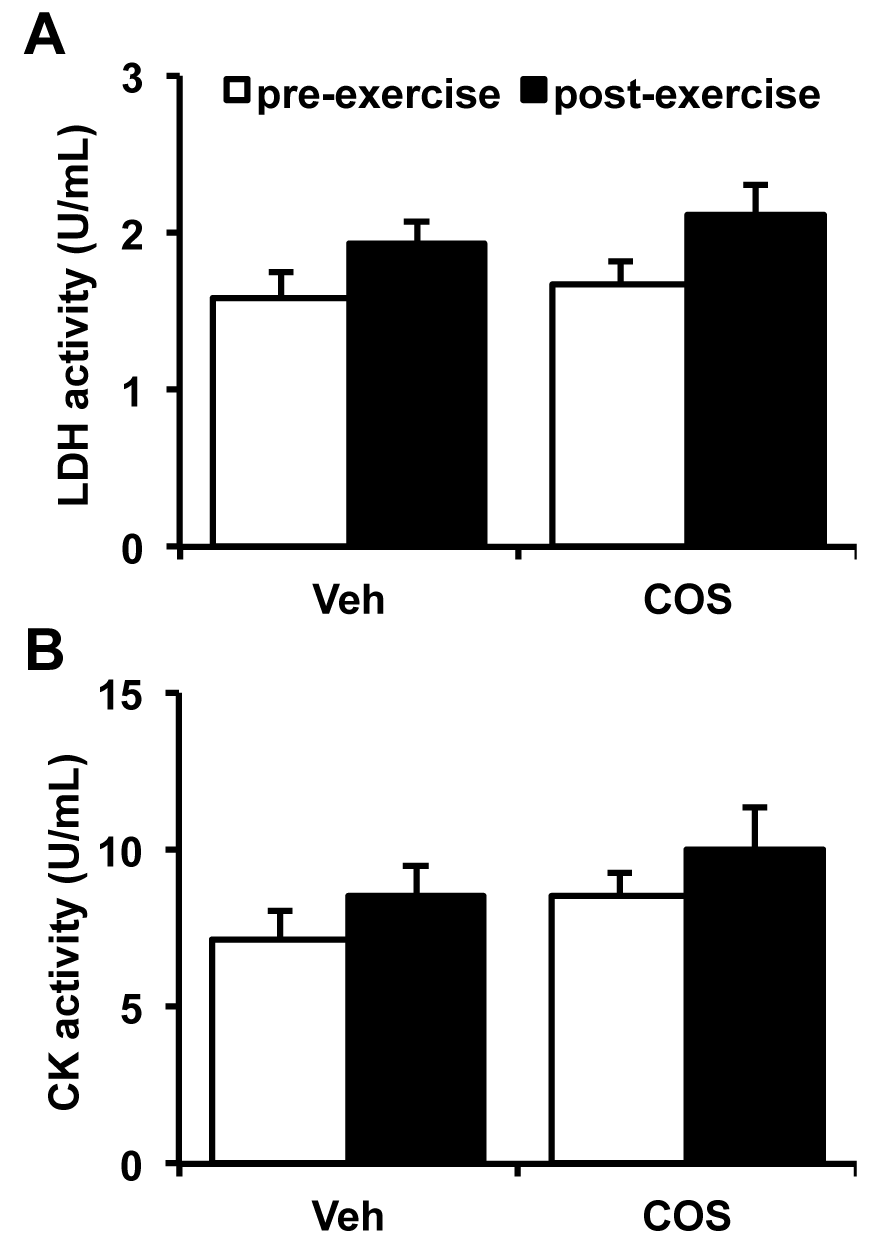

Supplement: Figure S5 — COS does not cause excess fatigue in spite of enhanced exercise endurance. Rats after endurance exercise were sacrificed and plasma LDH (A) and CK (B) activity was measured by using LDH and CK measuring kit (Bayer), respectively (n = 6). No statistical significance was observed among groups. (TIF) [file pone.0040073.s005.tif]

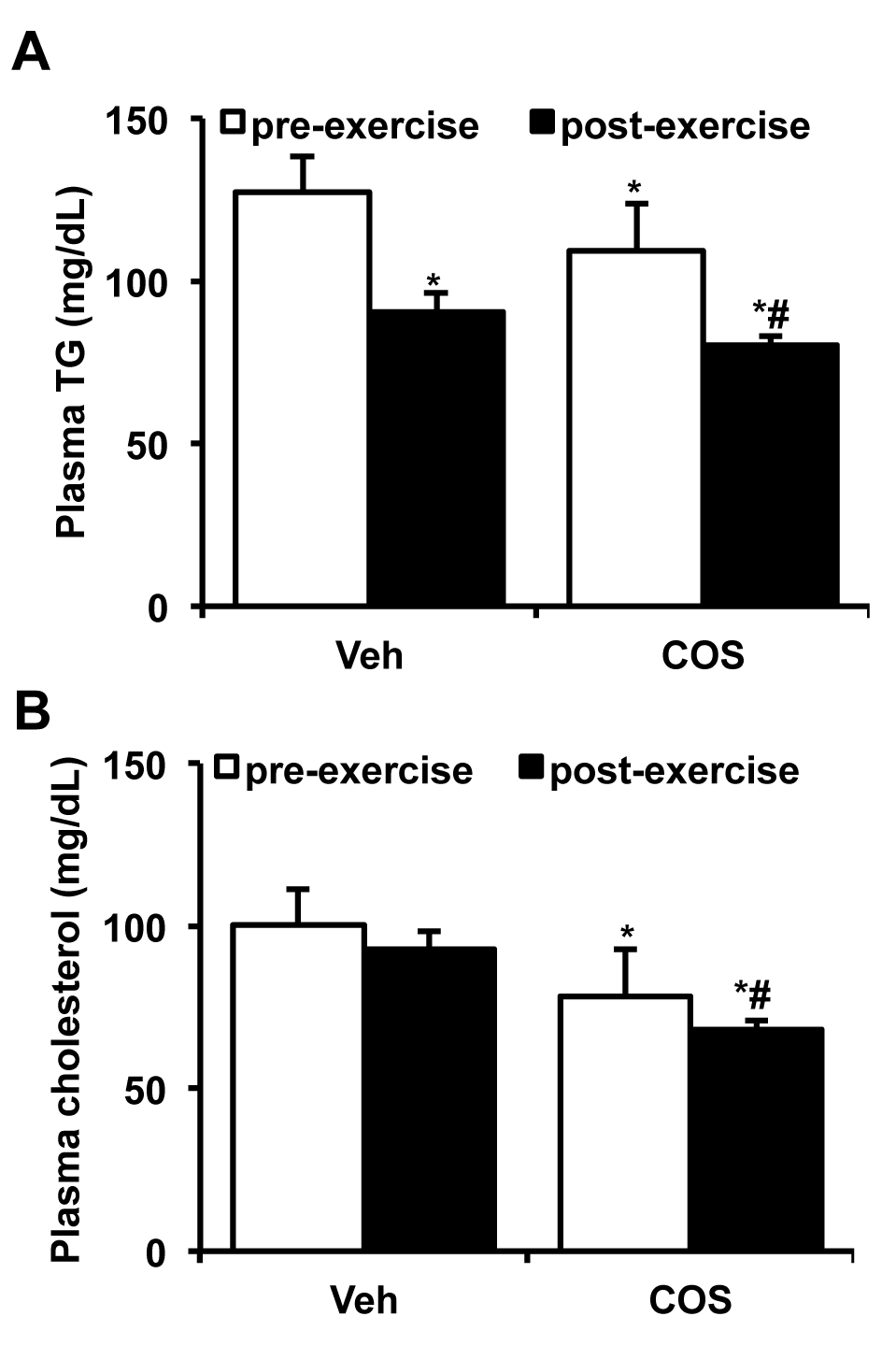

Supplement: Figure S6 — COS reduces plasma TG and cholesterol levels. Graphs show plasma profiles of TG (A) and cholesterol (B) of before (white bar) and after (black bar) exercise (n = 6). * P<0.05 vs. pre-exercise vehicle (lane 1); # P<0.05 vs. post-exercise vehicle (lane 2). (TIF) [file pone.0040073.s006.tif]

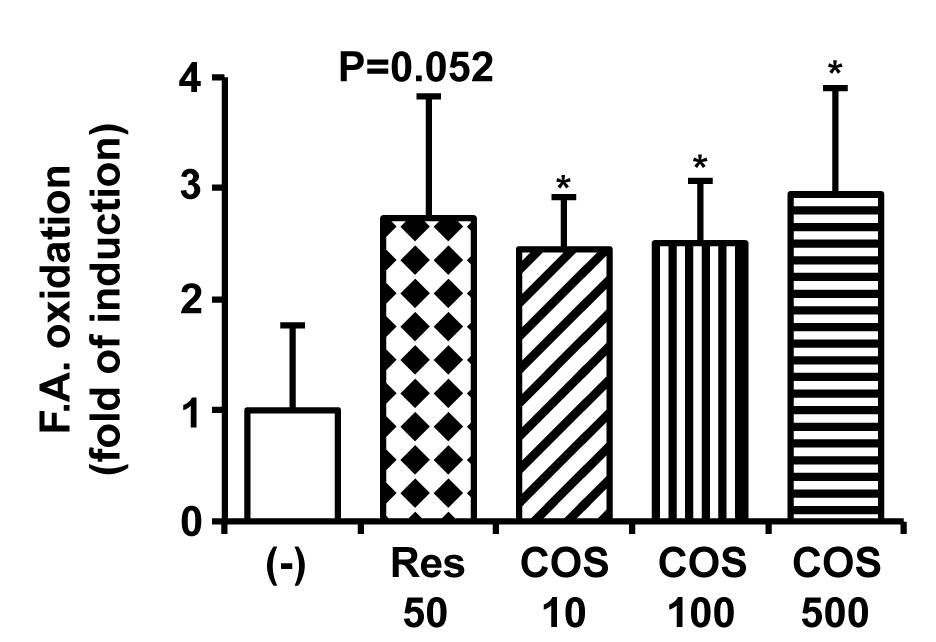

Supplement: Figure S7 — COS augments fatty acid oxidation. Differentiated C2C12 myocytes were treated with Res (11.4 µg/ml) or COS (10 µg/ml, 100 µg/ml, and 500 µg/ml) for 24 h (n = 10 in each group). After incubation, cells were rinsed with PBS and incubated in α-MEM (Lonza) containing 0.1 mmol/l palmitate (9,10-[3H]palmitate, 5 mCi/ml, PerkinElmer Life, Boston, MA) and 2% BSA for 24 h. The medium was then precipitated with an equal volume of 10% trichloroacetic acid (Sigma) by centrifugation at 12,000 rpm for 10 min. The supernatants were transferred to open 1.5 ml microcentrifuge tubes, placed in a scintillation vial containing 0.5 ml water, and incubated at 55°C for 12 h. After the removal of tubes containing the precipitated medium, the 3H2O contents were measured in a scintillation counter (PerkinElmer Life) in the presence of an enhancer solution (PerkinElmer Life). * P<0.05 vs. (-). (TIF) [file pone.0040073.s007.tif]

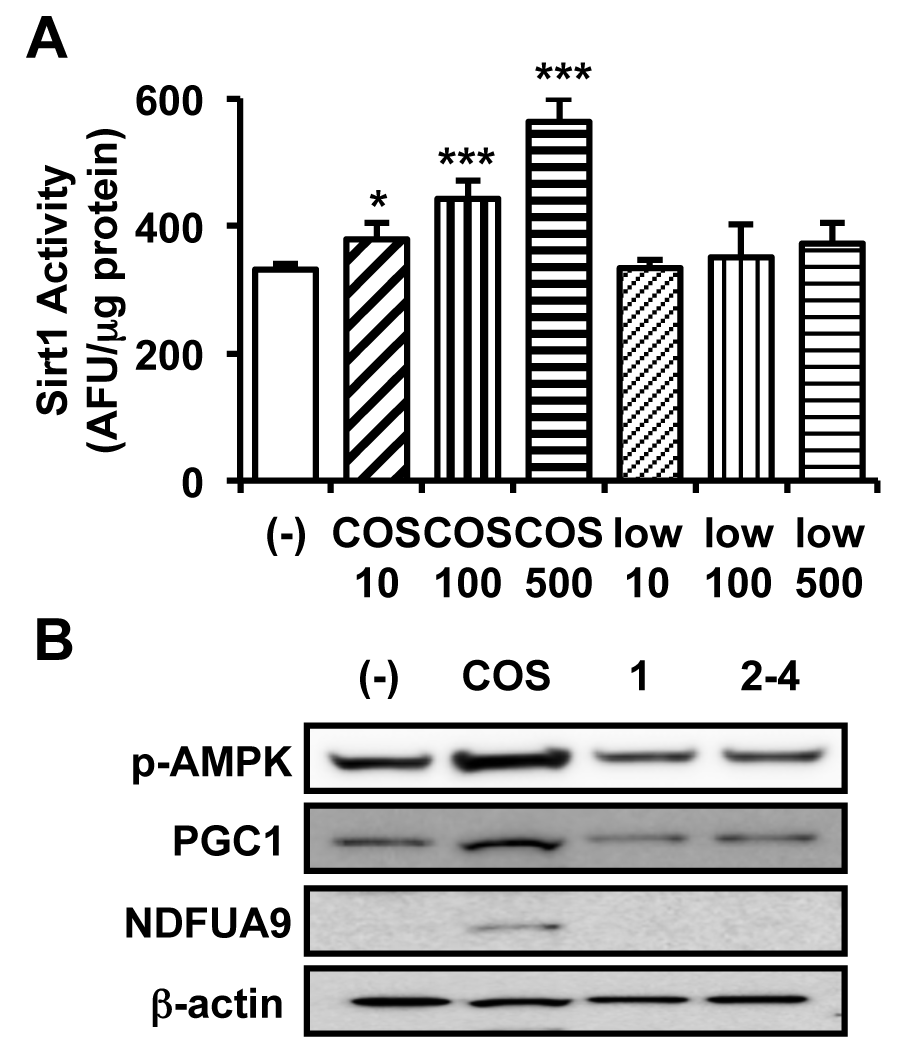

Supplement: Figure S8 — Small components of COS do not activate Sirt1 and AMPK. A. Sirt1 activity using small constituents of COS (LOW; monomer to tetramer) (n = 3). * P<0.05 vs. (-); ** P<0.01 vs. (-); *** P<0.001 vs. (-). B. Differentiated C2C12 myocytes were treated with COS (500 µg/ml), glucosamine-lactate (1; 500 µg/ml), or mixture of dimer, trimer, and tetramer (2–4; 500 µg/ml) for 24 hours. Proteins were separated and hybridized with p-AMPK, PGC1, NDUFA9, and β-actin antibodies respectively. The band intensity of each band is shown in Figure S9I. (TIF) [file pone.0040073.s008.tif]

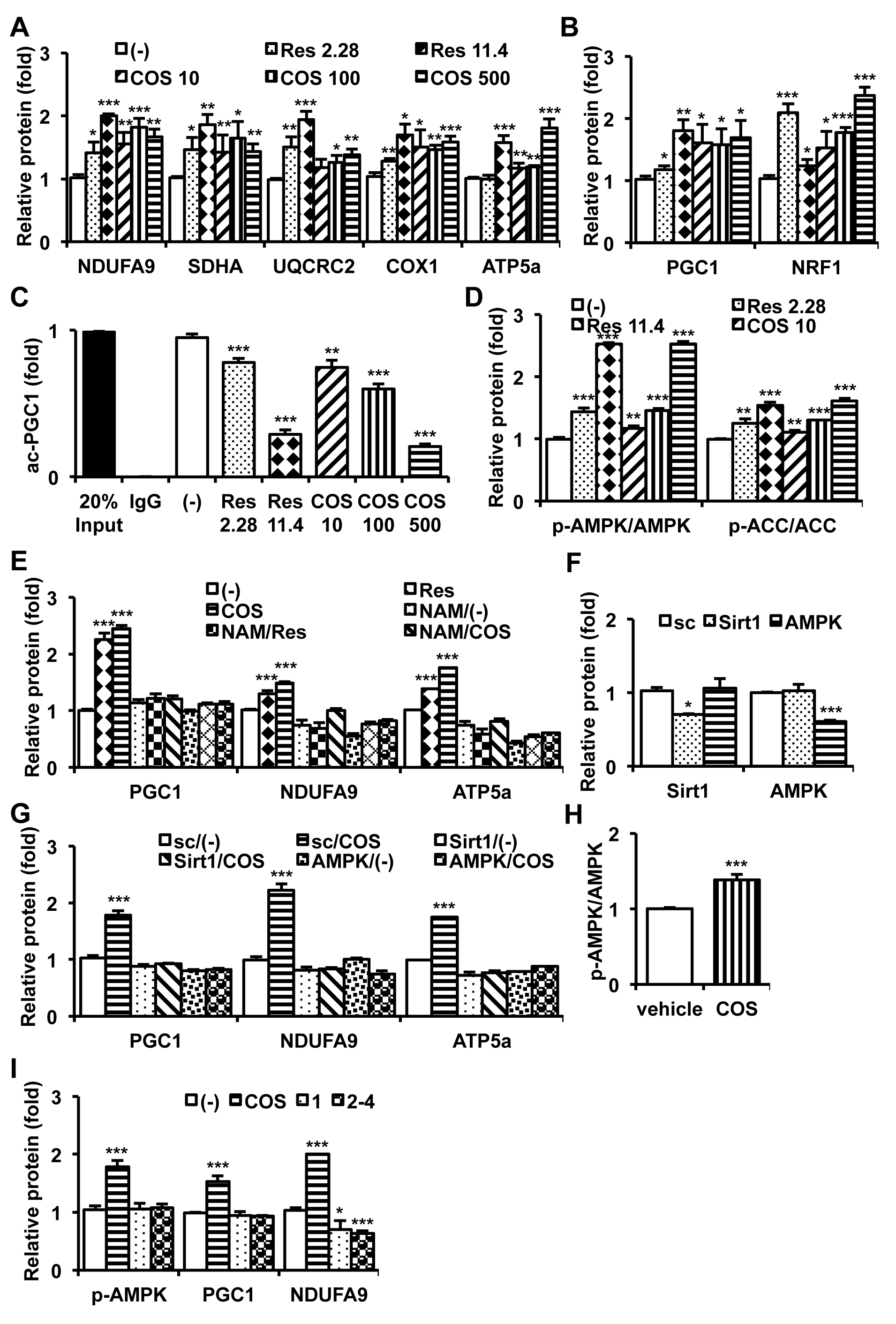

Supplement: Figure S9 — Densitometry of Western Blot results. Band intensity of each band in the Figure 2B (A), Figure 2C (B), Figure 3C (C), Figure 3D (D), Figure 4A (E), Figure S4B (F), Figure 5A (G), Figure 6B (H), and Figure S8B (I) was measured by Multiguage software (Fujifilm) and showed in bar graphs. * P<0.05 vs. (-); ** P<0.01 vs. (-); *** P<0.001 vs. (-). (TIF) [file pone.0040073.s009.tif]
