# Supplementary material for: Chitooligosaccharide Induces Mitochondrial Biogenesis and Increases Exercise Endurance through the Activation of Sirt1 and AMPK in Rats
Source: PLoS One. 2012 Jul 11;7(7):e40073. doi: 10.1371/journal.pone.0040073 (PMC3394803; doi:10.1371/journal.pone.0040073)
Supplement: Table S1 — Reaction condition of LC-MS/MS analysis. The composition of COS was analyzed by conducting LC-MS/MS analysis. The brief condition of analysis is shown. (DOC) [file pone.0040073.s010.doc]

**Table S1.** Reaction condition of LC-MS/MS analysis.

| A. | Agilent 6410 LC/MASS condition | |
| --- | --- | --- |
| Fragmentor | 150 | |
| Ion Source | ESI positive | |
| Gas Temp. (^○^C) | 320 | |
| Gas Flow (L/min) | 35 | |
| Capillary Voltage (V) | 4,000 | |
| B. | Agilent 1200 HPLC condition | |
| Column | U Bondapak NH2, 7.8 x 300 mm | |
| Flow Rate (mL/min) | 1.4 | |
| Injection amount (mg) | 2.0 | |
| Mobile Phase | A: 0.1% Formic acid in water, B: ACN | |
| Column Temp. (^○^C) | 35 | |
| C. | Agilent 1200 HPLC Gradient Condition | |
| Retention Time | Mobile phase composition | |
|  | A | B |
| 0 min | 20 | 80 |
| 5 min | 20 | 80 |
| 10 min | 65 | 35 |
| 15 min | 65 | 35 |
