# Supplementary material for: Chitooligosaccharide Induces Mitochondrial Biogenesis and Increases Exercise Endurance through the Activation of Sirt1 and AMPK in Rats
Source: PLoS One. 2012 Jul 11;7(7):e40073. doi: 10.1371/journal.pone.0040073 (PMC3394803; doi:10.1371/journal.pone.0040073)
Supplement: Table S2 — Sequences of qPCR primers. The qPCR primers for mitochondria-related genes (PGC1α, PGC1β, NRF1, tfam, and CPT1b) were used to examine the effect of COS on mitochondrial biogenesis. The qPCR primers for AMPKα1 and Sirt1 were used to determine the knock-down efficiency of AMPKα and Sirt1 siRNAs, respectively. GAPDH primer was used to normalize the relative mRNA expression of each gene. All qPCR primers were purchased from Bioneer Co. (DOC) [file pone.0040073.s011.doc]

**Table S2.** Sequences of qPCR primers.

| Gene | Forward | Reverse |
| --- | --- | --- |
| GAPDH | TGCACCACCAACTGCTTAG | GGATGCAGGGATGATGTTC |
| PGC1α | CCTCCTCATAAAGCCAACCA | GGGCCGTTTAGTCTTCCTTT |
| PGC1β | AACCCAACCAGTCTCACAGG | CTCCTAGGGGCCTTTGTTTC |
| NRF1 | TGATCCTGGAAGACCTCGAG | CTCGAGGTCTTCCAGGATCA |
| Tfam | CCAAAAAGACCTCGTTCAGC | ATGTCTCCGGATCGTTTCAC |
| CPT1b | TGTCATGGCAACAGTTGGTT | GACTCCGGTGGAGAAGATGA |
| AMPKα1 | ACCAGGACCCACTAGCCGTCG | GAGTCAGGTGGGCTGGTTGCT |
| Sirt1 | AGTGGCACATGCCAGAGTCCAA | CCAGCACATTCGGGCCTCTCC |
